# The ER Membrane Protein Complex Promotes Biogenesis of Dengue and Zika Virus Non-structural Multi-pass Transmembrane Proteins to Support Infection

**DOI:** 10.1016/j.celrep.2019.04.051

**Published:** 2019-05-07

**Authors:** David L. Lin, Takamasa Inoue, Yu-Jie Chen, Aaron Chang, Billy Tsai, Andrew W. Tai

**Affiliations:** 1 Department of Microbiology and Immunology, University of Michigan Medical School, Ann Arbor, MI 48109, USA; 2 Department of Cell & Developmental Biology, University of Michigan Medical School, Ann Arbor, MI 48109, USA; 3 Infectious Pathogen Research Section, Central Research Laboratory, Research and Development Division, Japan Blood Products Organization, Kobe 650-0047, Japan; 4 Division of Gastroenterology, Department of Internal Medicine, University of Michigan Medical School, Ann Arbor, MI 48109, USA; 5 Medicine Service, VA Ann Arbor Healthcare System, Ann Arbor, MI 48105, USA; 6 These authors contributed equally; 7 Lead Contact

## Abstract

Although flaviviruses co-opt the function of the host endoplasmic reticulum (ER) membrane protein complex (EMC) during infection, a mechanistic explanation for this observation remains unclear. Here, we show that the EMC promotes biogenesis of dengue virus (DENV) and Zika virus (ZIKV) non-structural multi-pass transmembrane proteins NS4A and NS4B, which are necessary for viral replication. The EMC binds to NS4B and colocalizes with the DENV replication organelle. Mapping analysis reveals that the two N-terminal marginally hydrophobic domains of NS4B confer EMC dependency. Furthermore, altering the hydrophobicity of these two marginally hydrophobic domains relieves NS4B’s EMC dependency. We demonstrate that NS4B biogenesis, but not its stability, is reduced in EMC-depleted cells. Our data suggest that the EMC acts as a multi-pass transmembrane chaperone required for expression of at least two virally encoded proteins essential for flavivirus infection and point to a shared vulnerability during the viral life cycle that could be exploited for antiviral therapy.

## In Brief

Multiple genetic screens have identified the ER membrane protein complex (EMC) as essential for infection by dengue and Zika flaviviruses. Lin et al. demonstrate that efficient biogenesis of the viral non-structural proteins NS4A and NS4B requires the EMC.

## Graphical Abstract

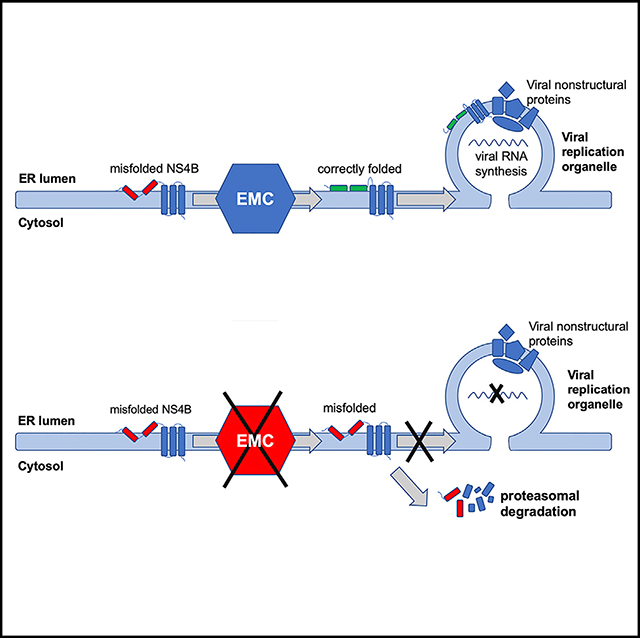

## INTRODUCTION

Dengue virus (DENV) is the most prevalent arboviral disease globally, with up to 400 million infections and 25,000 deaths annually (Bhatt et al., 2013). Similarly, the related flavivirus Zika virus (ZIKV) has spread rapidly across the tropics and subtropics, with outbreaks of DENV and ZIKV now reaching the continental United States. There are no effective antiviral treatments and no vaccine approved for use in the United States for either of these viruses.

All flaviviruses share a common genetic organization in which the positive-strand RNA genome encodes a single polyprotein that is translated at the endoplasmic reticulum (ER) and processed by host and viral proteases into ten viral structural and non-structural (NS) proteins. These NS proteins remodel the ER to form virus-induced membrane invaginations where genome replication occurs (Cortese et al., 2017; Welsch et al., 2009). Not surprisingly, multiple independent genetic screens have identified several cellular ER multiprotein complexes as dependency factors for flavivirus infection (Lin et al., 2017; Marceau et al., 2016; Savidis et al., 2016; Zhang et al., 2016). One of these complexes, the ER membrane protein complex (EMC), has been proposed to function as an ER chaperone for multi-pass transmembrane proteins (Jonikas et al., 2009; Richard et al., 2013; Satoh et al., 2015; Shurtleff et al., 2018), as well as an insertase for selective tail-anchored membrane proteins (Guna et al., 2018). In addition to being necessary for flavivirus infection, polyomavirus SV40 entry depends on the EMC (Bagchi et al., 2016). Four of the NS proteins (NS2A, NS2B, NS4A, and NS4B) are multi-pass transmembrane proteins; whether cellular mechanisms exist to promote the expression, folding, and stability of these proteins is unknown. Unstable or misfolded ER proteins are targeted by the ER-associated degradation (ERAD) pathway for ubiquitination and retrotranslocation into the cytosol for subsequent proteasomal degradation (Wu and Rapoport, 2018). Here we demonstrate that the NS4A and NS4B proteins of both DENV and ZIKV require the EMC for optimal expression. Furthermore, we demonstrate that dependence of NS4B on the EMC requires the presence of two weakly hydrophobic N-terminal helices. These results reveal a common dependence of two flaviviruses on the EMC through stabilization of two multi-pass transmembrane proteins and point to a shared vulnerability that could potentially be exploited as a broadly antiviral strategy.

## RESULTS

### The EMC Is Necessary for DENV Replication

**Figure 1.**
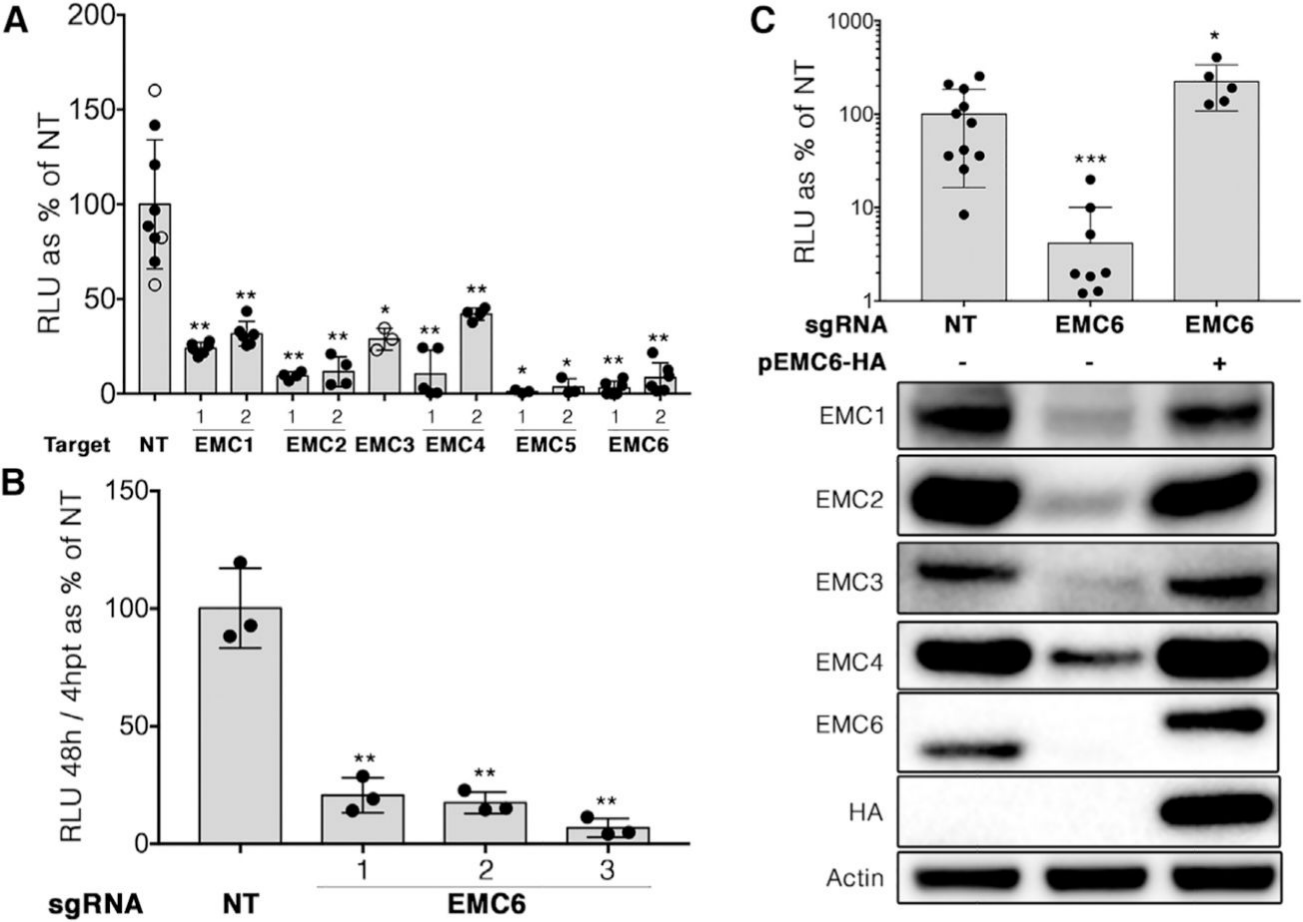
DENV Requires the EMC for Replication Huh 7.5.1 cells were stably transduced with pLentiCRISPRv2 lentiviral vectors encoding Cas9 nuclease and a targeting sgRNA to knock out the indicated gene or a non-targeting (NT) sgRNA control against GFP. (A) Filled circles: two independent sgRNAs were used per gene. Cells were then infected with a luciferase reporter DENV (luc-DENV), and luciferase activity was measured 3 days post-infection as relative light units (RLU). Open circles: for EMC3, cells were transfected with either EMC3 siRNA or a scrambled negative control siRNA. Two days after transfection, cells were then infected with luc-DENV, and luciferase activity was measured 3 days post-infection. (B) Cells expressing three different sgRNAs targeting EMC6 or a NT control were transfected with *in vitro* transcribed RNA encoding a luciferase reporter DENV replicon. Replication was assessed by the ratio between luciferase activity at 48 versus 4 h post-transfection to control for differences in transfection and translation efficiency. (C) EMC6 knockout cells were transduced to express an sgRNA-resistant HA tagged EMC6, then infected with luc-DENV, and luciferase activity was measured 3 days post-infection. Duplicate wells were lysed for immunoblotting with the indicated antibodies. Data are plotted as relative luciferase units (RLU) as a percentage of NT. Each dot represents a biological replicate, and bars show mean ± SD relative to cells expressing a non-targeting sgRNA control. Statistical significance compared with NT was determined using the Mann-Whitney U test (*p < 0.05, **p < 0.005, and ***p < 0.0005). See also Figures S1 and S2.

The six core subunits of the EMC, EMC1-EMC6, were identified as host dependency factors for flavivirus infection in four independent screens (Lin et al., 2017; Marceau et al., 2016; Savidis et al., 2016; Zhang et al., 2016). We validated that these EMC subunits were indeed necessary for DENV infection by first generating pooled EMC knockout Huh 7.5.1 cells using CRISPR/Cas9 technology. We found that knockout cells lacking EMC subunit 1, 2, 4, 5, or 6 were significantly reduced in their ability to support DENV infection compared with wild-type control cells (Figure 1A, filled circles). Because EMC3 knockout by CRISPR/Cas9 was poorly tolerated by Huh 7.5.1 cells, we used small interfering RNA (siRNA) knockdown to demonstrate that EMC3 depletion also inhibits DENV infection (Figure 1A, open circles).

Using a transient replication assay with a luciferase reporter DENV subgenomic replicon, we found that EMC6 knockout cells supported significantly reduced levels of DENV replication (Figure 1B). These data corroborate previous findings, suggesting that EMC depletion inhibits the initial stages of replication (Savidis et al., 2016). In contrast, hepatitis C virus (HCV) infection does not require the EMC (Figure S1), demonstrating specificity of viral inhibition by EMC knockout.

We also confirmed that long-term EMC6 depletion leads to the loss of other EMC subunits (Figure 1C; Guna et al., 2018; Tang et al., 2017) without affecting their mRNA levels (Figure S2), suggesting that EMC6 is required for the stability of the other EMC subunits. Exogenous expression of a single guide RNA (sgRNA)-resistant EMC6 construct in EMC6 knockout cells fully restored their ability to support DENV infection, as well as the expression of the other EMC subunits (Figure 1C). Collectively, these results demonstrate that the EMC is required to support DENV infection.

### The EMC Is Necessary for Expression of Flavivirus NS4A and NS4B by Promoting Their Biogenesis

**Figure 2.**
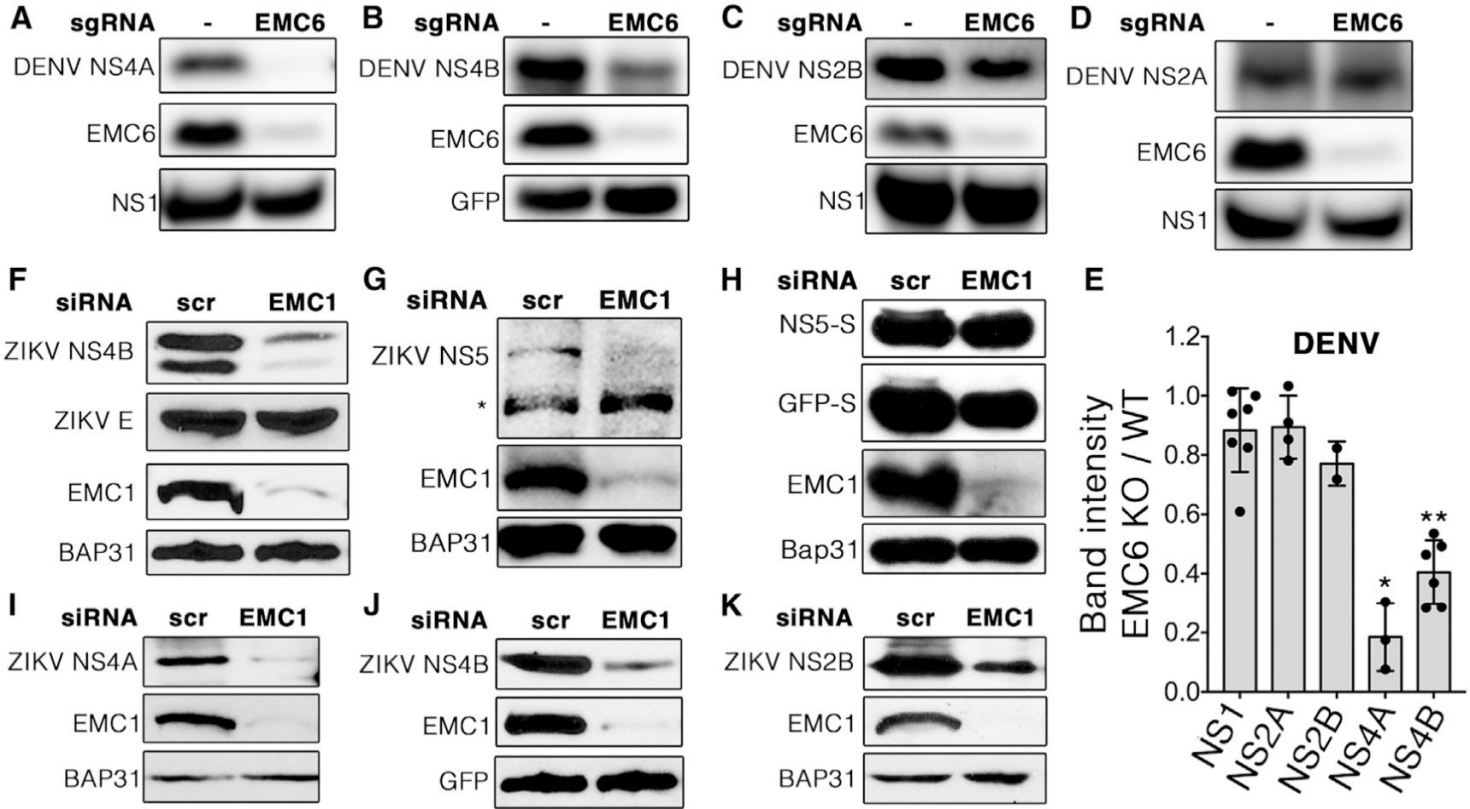
Flavivirus NS4A and NS4B Are Dependent on the EMC for Efficient Expression (A–D) 293T cell pools stably expressing Cas9 nuclease and an sgRNA targeting EMC6 were subsequently transiently co-transfected with constructs encoding HA-tagged DENV non-structural proteins and NS1-FLAG (A, C, and D) or GFP (B) as a transfection control. (E) ImageJ (NIH) was used to quantify band intensities for western blots of each of the DENV non-structural proteins in EMC6 knockout cells compared with wild-type from (A)–(D). Each dot represents a biological replicate. Bars represent mean ± SD. The Mann-Whitney U test was performed to assess statistical significance (*p < 0.05 and **p < 0.005 compared to NS1). (F–K) HEK293 cells were transfected with siRNAs against EMC1 or a scrambled negative control (scr). Forty-eight hours later, cells were transfected with a replication-defective full-length ZIKV cDNA (F and G) or with plasmids encoding individual S-tagged ZIKV non-structural proteins NS5 (H), NS4A (I), NS4B (J), or NS2B (K). The asterisk in (G) indicates a nonspecific background band. For (A)–(D) and (F)–(K) 24 h post-transfection, cells were lysed and proteins were separated using SDS-PAGE followed by western blotting for the indicated proteins. Each blot is representative of a minimum of two biological replicates. See also Figure S3.

The EMC has been shown to act as a multi-pass transmembrane chaperone (Jonikas et al., 2009; Richard et al., 2013; Satoh et al., 2015) and has also been proposed to function as a transmembrane insertase (Guna et al., 2018). As the DENV genome encodes several multi-pass transmembrane NS proteins (NS2A, NS2B, NS4A, and NS4B), we hypothesized that the EMC is necessary for biogenesis of one or more of these NS proteins. To test this, we transfected wild-type and EMC knockout cells to individually express each of the multi-pass transmembrane DENV NS proteins and assessed the steady-state expression level of each of the proteins by immunoblotting. Strikingly, steady-state expression of both DENV NS4A and NS4B was significantly reduced in EMC knockout cells compared with wild-type cells (Figures 2A and 2B; quantified in Figure 2E). By contrast, expression of NS2B was moderately decreased in EMC knockout cells compared with wild-type cells (Figure 2C; quantified in Figure 2E), while NS2A expression was unaffected by the loss of the EMC (Figure 2D; quantified in Figure 2E). As expected, expression of either soluble NS1 or GFP was also unaffected by the loss of the EMC (Figures 2A–2E).

To generalize these findings to other flavivirus family members, we performed similar experiments for ZIKV NS proteins. As with EMC6 knockout, pronounced EMC1 knockdown in HEK293T cells by siRNA also led to depletion of the other EMC subunits (Figure S3). We next transfected wild-type (scrambled siRNA-treated) and EMC1 knockdown cells to express a full-length replication-defective ZIKV polyprotein and observed a similar reduction in the steady-state expression level of NS4B in EMC knockdown cells (Figure 2F). Intriguingly, although expression of the viral E protein from the amino-terminal end of the same polyprotein was unaffected by loss of the EMC, despite also being a multi-pass transmembrane protein (Figure 2F), steady-state expression of the C-terminal NS5 protein was diminished in EMC-depleted cells when NS5 was expressed from the full-length ZIKV polyprotein (Figure 2G). Contrary to the reduction of NS5 expression when expressed from the same polyprotein as NS4A and NS4B in EMC-depleted cells, ZIKV NS5 levels were unaffected by EMC depletion when expressed in isolation (Figure 2H). However, expression of both NS4A and NS4B was significantly reduced in EMC knockdown cells, even when these proteins were expressed in isolation (Figures 2I and 2J), whereas expression of transfected GFP or the endogenous multi-pass ER membrane protein BAP31 was unaffected by EMC1 depletion. In contrast to DENV NS2B, expression of ZIKV NS2B was also markedly decreased in EMC1-silenced cells (Figure 2K), suggesting a difference in EMC dependency for this NS protein.

**Figure 3.**
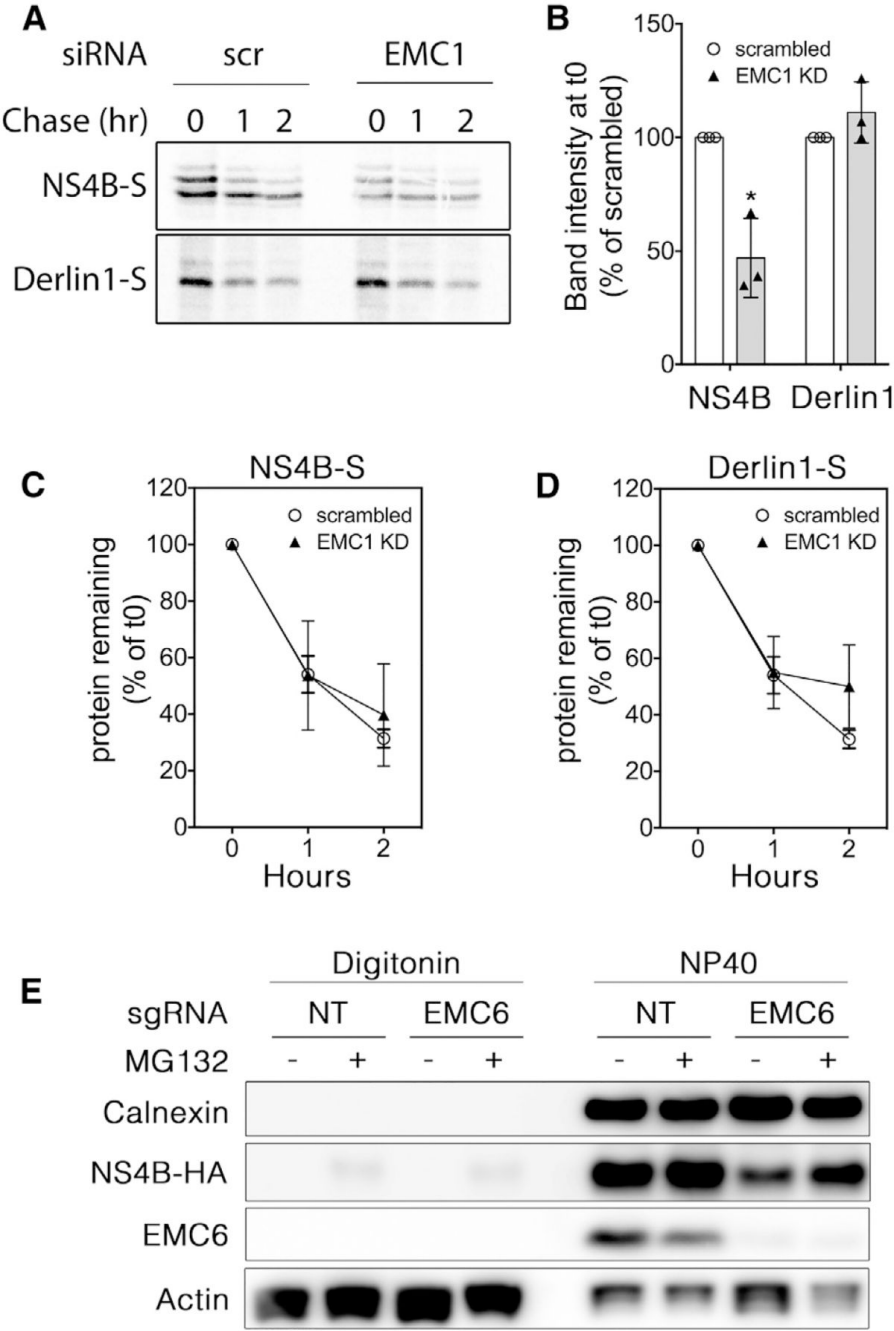
The EMC Is Required for NS4B Biogenesis but Not for Post-translational Stability (A) HEK293T cells were transfected with scrambled or EMC1 siRNAs followed by transfection with S-tagged ZIKV NS4B or Derlinl. Twenty-four hours post-transfection, cells were pulse-labeled with [^35^S]Met/Cys for 20 min and chased for the indicated times. NS4B-S or Derlin1-S was affinity-precipitated and subjected to SDS-PAGE, and the radiolabeled protein was visualized using phosphorimaging. Shown is a representative image from three biological replicates. (B) The protein levels of NS4B-S and Derlin1-S at t = 0 were quantified by phosphorimaging. Data from three biological replicates are plotted as mean ± SD. (C and D) Levels of labeled NS4B-S protein (C) or Derlin1-S protein (D) in cells transfected with EMC1 siRNA (closed triangles) or negative control scrambled siRNA (open circles) were quantified at the indicated chase times and normalized to the levels at t = 0 in order to determine the degradation rate after biogenesis. Data from three biological replicates are plotted as mean ± SD. (E) Wild-type or EMC6 knockout Huh 7.5.1 cells were stably transduced to express NS4B-HA, then treated with 10 μM MG132 for 3 h to inhibit proteasomal activity. Cells were then treated with 0.02% digitonin to permeabilize the plasma membrane and release cytoplasmic proteins. The digitonin-soluble supernatant was collected, and then the digitonin-insoluble material was subjected to 1% NP40 treatment to release ER luminal proteins. The fractions were separated using SDS-PAGE and visualized by western blotting. Shown is a representative blot from two independent experiments.

The observed decrease in steady-state levels of NS4A and NS4B in EMC-depleted cells could be due to a decrease in protein biogenesis and/or a decrease in protein stability. To resolve these two possibilities, we performed a metabolic pulse-chase analysis of ZIKV NS4B and of the ER transmembrane protein Derlin1 in cells transfected with an EMC1 siRNA or a scrambled negative control siRNA. Levels of newly synthesized NS4B following a 20 min pulse were significantly diminished in EMC1-silenced cells compared with control cells (Figure 3A), whereas levels of Derlin1 were unaffected; the initial protein levels of NS4B and Derlin1 before chase (i.e., T0) in control and EMC1-silenced cells are quantified in Figure 3B. Moreover, during a chase period of up to 2 h, we detected no significant change in the half-life of newly synthesized NS4B (Figure 3C) or Derlin1 (Figure 3D), indicating that the stability of these proteins after biogenesis is not dependent on the EMC.

We next tested whether the EMC is required for membrane association of NS4B using a cell permeabilization assay because the EMC was reported to promote membrane insertion of a subset of tail-anchored proteins (Guna et al., 2018). The decrease in NS4B expression in EMC-depleted cells can be partially restored by proteasomal inhibition (Figure 3E, right lanes). Wild-type or EMC6 knockout cells were transfected to express NS4B-HA with or without proteasome inhibition with MG132 to stabilize NS4B. We then subjected the cells to detergent treatment with NP-40, which solubilizes the plasma and ER membranes, or with digitonin, which at low concentrations selectively permeabilizes the plasma membrane but not the ER membrane. As expected, the ER transmembrane proteins calnexin and NS4B were readily solubilized by NP-40, as well as ER-bound EMC6 and cytosolic actin (Figure 3E, right lanes). In contrast, digitonin permeabilization released only a very minor fraction of NS4B in MG132-treated wild-type or EMC6 knockout cells (Figure 3E, left lanes), indicating that the EMC is not required for ER membrane association of NS4B. Collectively, these data suggest that the EMC supports the biogenesis of flavivirus NS proteins, which are necessary for viral replication.

### Flavivirus NS4B Interacts with EMC and Translocon Subunits

**Figure 4.**
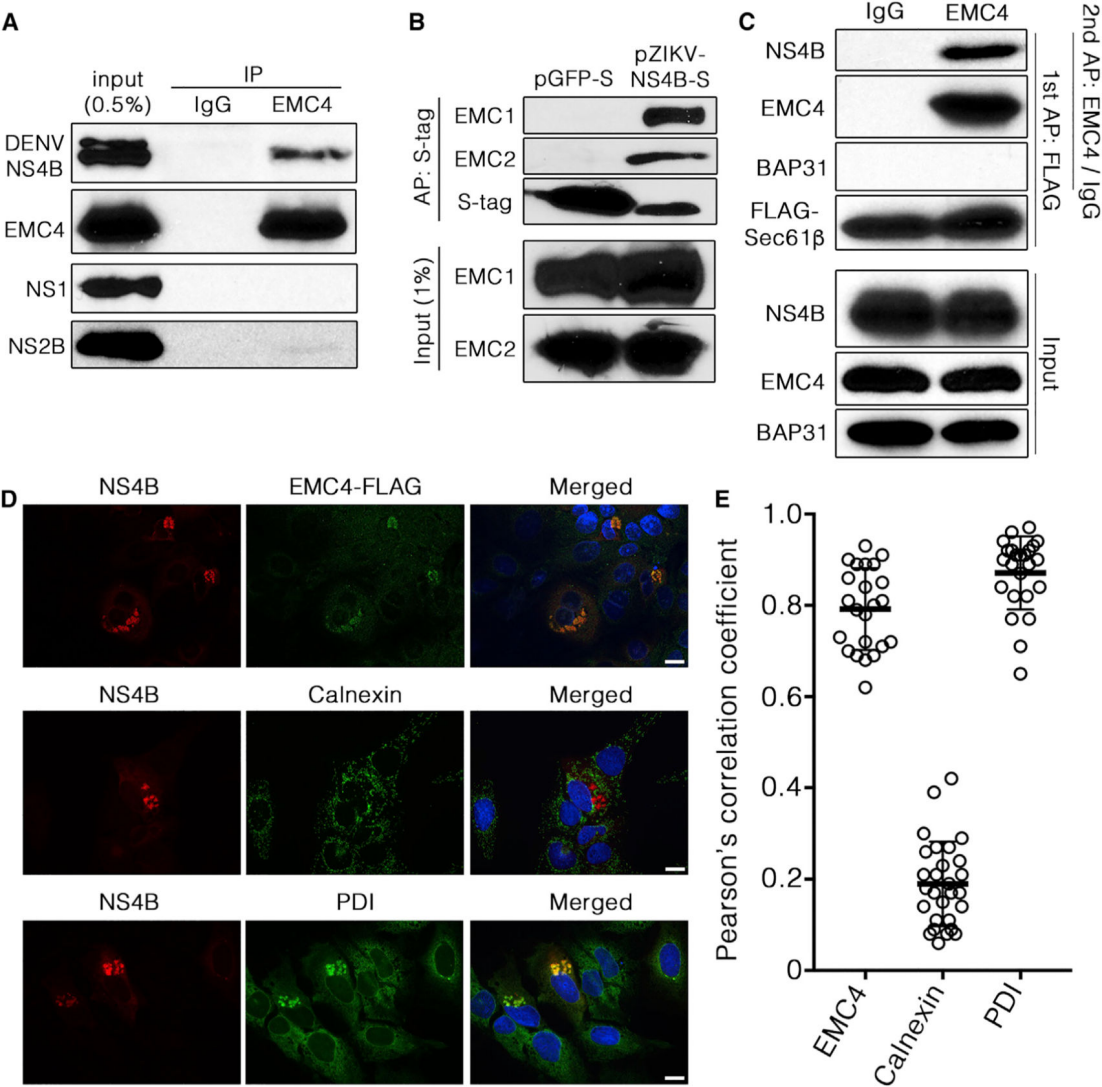
NS4B Interacts with EMC and Translocon Subunits (A) Huh 7.5.1 cells stably harboring a DENV replicon were lysed and subjected to Immunoprecipitation of endogenous EMC4 using an anti-EMC4 antibody or an isotype control. Immunoprecipitates were analyzed by immunoblotting with the indicated antibodies. Data are representative of three biological replicates. (B) 293 cells transiently transfected to express either GFP-S or ZIKV NS4B-S were lysed 24 h later for affinity purification by S-protein-conjugated beads and immunoblotting with the indicated antibodies. Data are representative of two biological replicates. (C) Huh 7.5.1 cells stably expressing a DENV replicon were transiently transfected with FLAG-Sec61β. Twenty-four hours post-transfection, cell lysates were prepared and subjected to anti-FLAG immunoprecipitation. The anti-FLAG immunoprecipitate underwent a second round of immunoprecipitation using an anti-EMC4 antibody. This second immunoprecipitate was subjected to SDS-PAGE followed by immunoblotting with the indicated antibodies. Data are representative of three biological replicates. (D) Stable DENV replicon Huh 7.5.1 cells were transduced to express EMC4-FLAG. Immunostaining was performed for the indicated antigens with DAPI nuclear counterstaining, followed by confocal microscopy. Scale bars, 10 μm. (E) Quantitation of colocalization was performed using Pearson’s coefficients; each point represents a single cell.

We next performed co-immunoprecipitation (coIP) experiments using an antibody against endogenous EMC4 in cells stably expressing a DENV replicon. Our results revealed that NS4B, but not NS1 or NS2B, significantly coIP with endogenous EMC4 (Figure 4A). In the reciprocal experiment, we found that affinity purification of transfected S-tagged ZIKV NS4B (but not the control S-tagged GFP) co-precipitated endogenous EMC1 and EMC2 (Figure 4B). These results demonstrate that the EMC binds to DENV and ZIKVNS4B.

Given our finding that NS4B biogenesis is inhibited by EMC depletion and by a previous report that the EMC interacts cotranslationally with substrate proteins (Shurtleff et al., 2018), we asked whether EMC interacts with NS4B at the ER translocon. We performed a sequential immunoprecipitation experiment in cells stably expressing a DENV replicon and a FLAG-tagged Sec61β translocon subunit by first immunoprecipitating FLAG-Sec61β, eluting bound proteins, and then immunoprecipitating EMC4 that had been bound to FLAG-Sec61β. As shown in Figure 4C, the immunoprecipitated material contained NS4B, consistent with a model in which NS4B interacts simultaneously with the translocon and with the EMC. This suggests that the EMC engages client multi-pass transmembrane proteins such as NS4B cotranslationally at the time of ER membrane translocation.

To further corroborate the physical interaction data, we asked if the EMC colocalizes with viral NS proteins in cells. To test this, we transduced DENV replicon cells to express FLAG-tagged EMC4 (EMC4-FLAG), then performed immunofluorescence staining and confocal microscopy to visualize localization of the EMC in DENV-infected cells. We found colocalization of EMC4-FLAG with puncta containing NS4B (Figure 4D, top row). By contrast, EMC4-FLAG was diffusely distributed in cells with little NS4B staining. Similarly, we found colocalization between NS4B and endogenous PDI (protein disulfide isomerase) but not between NS4B and endogenous calnexin (Figure 4D, compare third with second row), which is supported by quantitation of protein colocalization (Figure 4E). Collectively, the evidence supports a model in which the EMC is required for flavivirus infection by directly interacting with NS4B at the time of protein biogenesis to promote their expression.

### Mapping Determinants of EMC Dependence

**Figure 5.**
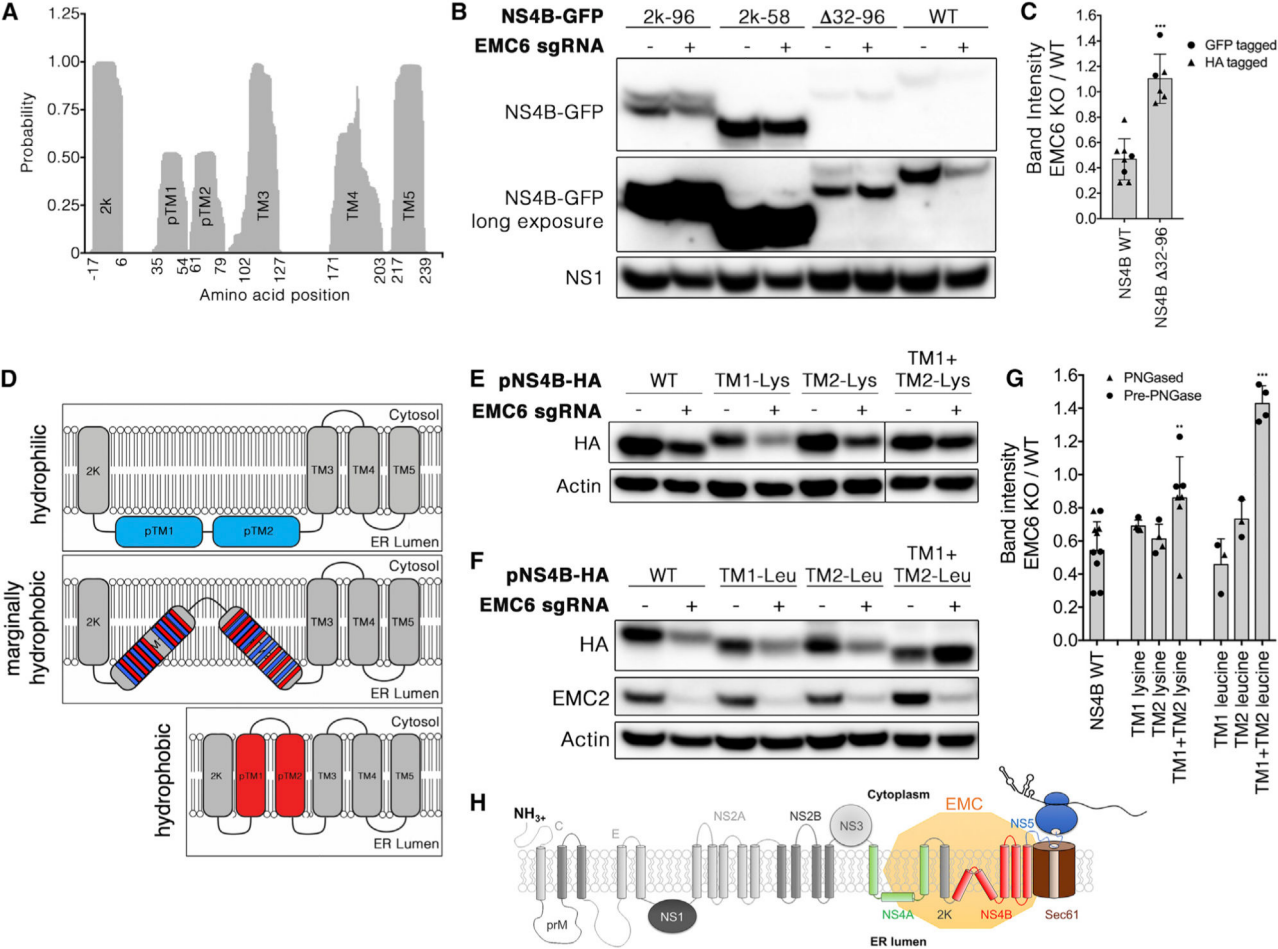
Identification of DENV NS4B Domains that Confer EMC Dependence (A) The TMHMM version 2.0 algorithm (Krogh et al., 2001)was used to predict the presence of transmembrane helices in 2k-NS4B of DENV (UniProtKB: P29990). This plot depicts the probability of a given residue being located within a transmembrane helix, where greater values reflect greater transmembrane probability. The indicated amino acid positions on the x axis indicate the boundaries of each predicted transmembrane domain with scores above 0.3 for each predicted transmembrane domain. (B) HEK293T cells stably expressing Cas9 and sgRNA targeting EMC6 were co-transfected to express the indicated C-terminally GFP-tagged NS4B mutants and NS1-FLAG as a transfection control. 2k-96 indicates a C-terminal truncation at amino acid 97. 2k-58 indicates a C-terminal truncation at amino acid 59. Δ32–96 indicates a deletion from 32 to 96. Twenty-four hours post-transfection, cells were lysed and proteins were resolved using SDS-PAGE followed by western blotting for the indicated proteins. (C) Band intensitiesforNS4Bwild-type and Δ32–96 in EMC6 knockout cells compared with wild-type cells. Each point represents a biological replicate from cells transfected with NS4B-GFP (circles) or NS4B-HA (triangles). (D) Schematic of NS4B mutations to alter hydrophobicity of the pTM1 and pTM2 helices. Both of these helices are speculated to be membrane associated, with red representing hydrophobic and blue representing charged and polar hydrophilic residues, respectively (middle). Mutagenesis of the hydrophobic residues to lysine (top) is expected to result in decreased membrane association of pTM1 and pTM2. Conversely, mutagenesis of charged or polar residues to leucine (bottom) is expected to result in the insertion of pTM1 and pTM2 into the membrane. (E and F) Wild-type 293T cells or cells stably expressing Cas9 and sgRNA targeting EMC6 were transfected to express the indicated NS4B-HA mutants with substitutions to lysine residues (less hydrophobic) shown in (E) and substitutions to leucine residues (more hydrophobic) shown in (F). Twenty-four hours post-transfection, cells were lysed and proteins were resolved using SDS-PAGE followed by western blotting for the indicated proteins. Blots are representative of a minimum of three independent experiments.(G) Bands were quantitated using ImageJ and represented as a ratio of band intensity in EMC6 knockout versus wild-type cells. Each point represents a biological replicate, with bars representing mean ± SD. Triangles represent quantification from blots of NS4B with PNGase treatment, while circles represent quantification from blots of NS4B without PNGase treatment. Statistical significance was assessed using Dunnett’s test for multiple comparisons (**p < 0.005 and ***p < 0.0005 compared with WT NS4B). (H) Model of EMC interaction with theSec61 translocon and the flavivirus protein. Depicted is a flavivirus genomic RNA (upper left) being translated by a ribosome (blue) at the cytosolic face of the ER. Transmembrane domains are cotranslationally inserted into the ER membrane by the Sec61 translocon (brown). The EMC (yellow) is associated with the translocon and assists in the proper insertion and stabilization of certain multi-pass transmembrane domain proteins at the time of protein translation and/or translocation. The expression of both NS4A and NS4B of DENV and ZIKV is dependent on the EMC. Expression of the next protein in the flavivirus polypeptide, NS5, is also decreased in EMC-deficient cells when NS5 is expressed on the same polyprotein as NS4A and NS4B but not when expressed by itself.

Given that NS4B engages the EMC, most likely at the time of protein biogenesis, we next sought to identify the specific determinants of NS4B that confer its dependence on the EMC. In addition to its postulated role as a molecular chaperone for multi-pass transmembrane proteins, the EMC also has been shown to function as an insertase for tail-anchored transmembrane proteins, particularly those with weakly hydrophobic transmembrane domains (Guna et al., 2018). Therefore, we hypothesized that weakly hydrophobic transmembrane domains in NS4A and NS4B might drive their EMC dependence. DENV NS4B harbors an N-terminal 2k signal peptide that is cotranslationally cleaved, followed by five predicted hydrophobic helices. Evidence suggests that the three C-terminal helices are membrane-spanning domains (Li et al., 2015; Miller et al., 2006). By contrast, transmembrane helix prediction by TMHMM reveals that the two N-terminal helices exhibit relatively low transmembrane probabilities compared with the three C-terminal helices (Figure 5A, pTM1 and pTM2); additionally, one report has suggested that these domains lie on the ER luminal membrane rather than spanning the lipid bilayer (Miller et al., 2006).

By analogy to tail-anchored transmembrane domains, we hypothesized that the two N-terminal weakly hydrophobic helices in NS4B are required for EMC dependence. To test this idea, we generated plasmids encoding a wild-type DENV 2k-NS4B-GFP fusion protein, a mutant lacking these two helices (Δ32–96) or harboring one or two of the N-terminal hydrophobic segments immediately following the 2k signal sequence (2k-58 and 2k-96). We co-transfected these plasmids along with NS1-FLAG as a transfection control into wild-type or EMC knockout cells and assessed their relative levels of expression by immunoblotting. Consistent with previous results, NS4B-GFP expression was significantly reduced in EMC knockout cells compared with wild-type cells (Figure 5B). However, expression of the NS4B (Δ32–96)-GFP mutant was unaffected by EMC knockout, indicating that loss of these helices rendered NS4B expression independent of the EMC (Figure 5B; quantified in Figure 5C). In contrast, neither NS4B (2k-96) nor NS4B (2k-58) displayed strong EMC dependency when fused to GFP (Figure 5B), suggesting that the N-terminal helices of NS4B are not sufficient for EMC dependence.

As another test of the hypothesis that the EMC dependence of NS4B is conferred by the weak hydrophobicity of the pTM1 and pTM2 helices, we generated NS4B mutants in which all of the hydrophobic residues in either or both of the two helices were exchanged for lysine residues or in which all of the polar and charged residues in either or both of the two helices were exchanged for hydrophobic leucine residues (Figure 5D). These constructs were transfected into wild-type or EMC6 knockout cells and their expression levels quantified after immunoblotting. Because fusion of NS4B to GFP could conceivably alter its dependence on the EMC, we used HA-tagged NS4B constructs for these experiments. As expected, wild-type NS4B-HA was also expressed at lower levels in EMC-depleted cells (Figures 5E and 5F, left two lanes; quantitated in Figure 5G). Although decreasing the hydrophobicity of either pTM1 or pTM2 did not significantly increase NS4B expression in EMC6-depleted cells, decreasing the hydrophobicity of both significantly increased NS4B expression in EMC6-depleted cells (Figures 5E and 5G). Conversely, although increasing the hydrophobicity of either pTM1 or pTM2 had no or moderate effects on EMC dependence, respectively, increasing the hydrophobicity of both resulted in an NS4B protein that was completely insensitive to EMC knockout (Figures 5F and 5G). Thus, our results are consistent with the idea that the marginally hydrophobic character of the two N-terminal NS4B helices is required for EMC dependence.

## DISCUSSION

The infection cycles of flaviviruses depend on the ER. Flavivirus infection induces extensive remodeling of the ER membrane to form specialized replication organelles (Cortese et al., 2017; Welsch et al., 2009). These structures have been proposed to shield viral products, such as double-stranded RNA, from innate immune recognition, and to concentrate factors that promote viral replication. We speculate that another function of these organelles is to generate a specialized environment for polyprotein translation, processing, and folding. The viral polyprotein is translated and processed at the ER, and it has been shown that the cytosolic capsid and NS5 proteins of DENV depend on Hsp70 chaperones for their stability (Taguwa et al., 2015). However, whether flaviviral membrane proteins also require cellular chaperones for their proper expression, folding, and stability remains unknown.

The EMC has been identified as a host dependency factor for infection by the flaviviruses DENV, ZIKV, and yellow fever virus (YFV) (Lin et al., 2017; Marceau et al., 2016; Savidis et al., 2016; Zhang et al., 2016). Although the functions of the EMC remain incompletely understood, accumulating evidence indicates that it acts as an ER-localized molecular chaperone for a subset of multi-pass transmembrane proteins (Jonikas et al., 2009; Richard et al., 2013; Satoh et al., 2015), as well as a tail-anchored transmembrane protein insertase (Guna et al., 2018). As the EMC likely acts as a chaperone for multiple cellular membrane proteins, we cannot exclude the possibility that a cellular membrane protein essential for DENV and ZIKV infection is not stably expressed or misfolded in EMC-depleted cells. For example, it has been suggested that the EMC is required for expression of a ZIKV entry factor (Savidis et al., 2016). However, this does not exclude a role for the EMC in NS4A and NS4B biogenesis. In fact, several lines of evidence argue that the EMC functions as a molecular chaperone for these viral proteins (Figure 5H): (1) both are multi-pass transmembrane proteins expressed on the ER and thus are biologically plausible substrates; (2) the levels of both proteins are specifically reduced by EMC depletion and restored by proteasome inhibition; and (3) an NS4B-EMC-translocon interaction was identified by sequential coIP analysis. By pulse-chase analysis, we found that the biogenesis of NS4B was inhibited in EMC-depleted cells; however, the fraction of NS4B that was synthesized despite EMC depletion displayed a similar half-life as NS4B synthesized in wild-type cells, indicating that stability of NS4B after translation and translocation into the ER membrane does not require the EMC.

In addition to the decreased expression of NS4A and NS4B in EMC knockout cells, we also found that ZIKV NS5 steady-state expression levels were significantly reduced in EMC-depleted cells when expressed from a full-length ZIKV polyprotein but not when expressed in isolation. The NS5 protein is located C-terminal to NS4Aand NS4B in the flavivirus genome, suggesting that in the absence of the EMC, the biogenesis of the flavivirus protein downstream of NS4A and NS4B is impaired. In contrast, the level of the ZIKV E protein, which is N-terminal to NS4A and NS4B, is unaffected by EMC depletion when expressed from a full-length ZIKV polyprotein.

Although DENV NS2B was relatively unaffected by EMC knockout, expression of ZIKV NS2B was significantly reduced. These data suggest the possibility that the EMC may be additionally required for expression of different NS proteins for each flavivirus. Furthermore, HCV does not depend on the EMC for infection despite encoding several multi-pass transmembrane proteins. This specificity invokes the obvious question: what are the determinants within multi-pass transmembrane proteins that drive EMC dependence? We showed that NS4B can be made to be EMC independent either by deletion of two marginally hydrophobic helices in NS4B or by altering the hydrophobicity of both helices, consistent with the proposal that the EMC recognizes weakly hydrophobic segments in both multi-pass transmembrane and tail-anchored membrane proteins.

Our findings regarding EMC function are conceptually in agreement with a recent publication (Shurtleff et al., 2018) concluding, on the basis of ribosome labeling and proteomics in yeast and human cells, that the EMC serves to stabilize multi-pass transmembrane proteins encoding transmembrane domains enriched for charged residues. However, there was no experimental demonstration that mutation of any putative EMC client could modulate its EMC dependence. They also identified a surprisingly limited number of cellular proteins that displayed significant EMC dependence: only 11 proteins were decreased by 2-fold or more in both EMC2 and EMC4-depleted human cells. Our work adds substantially to these findings by the experimental demonstration that the marginally hydrophobic segments of NS4B are required for stabilization by the EMC. Furthermore, the NS4A and NS4B proteins of both ZIKV and DENV display strong dependence on the EMC for their expression, which is consistent with cells being able to tolerate genetic depletion of EMC subunits while these flaviviruses are not.

In summary, this work defines a mechanism by which the EMC supports flavivirus replication and provides additional evidence that the EMC functions as a multi-pass transmembrane chaperone. The dependence of multiple flaviviruses on the EMC and on Hsp70 proteins highlights their dependence on ER quality control mechanisms and therefore a shared vulnerability that potentially could lead to broadly antiviral strategies.

## STAR⋆METHODS

### CONTACT FOR REAGENT AND RESOURCE SHARING

Further information and requests for reagents and resources should be directed to and will be fulfilled by the Lead Contact, Andrew Tai (andrewwt@umich.edu). The DENV-2 virus strain 16681 and its derivative Luc-DENV are covered by an MTA with the Centers of Disease Control (CDC); the anti-DENV NS1 clone 1F11 antibody is covered by an MTA with the National Science and Technology Development Agency, Thailand.

### EXPERIMENTAL MODEL AND SUBJECT DETAILS

#### Cell lines

293T and the derivative line Flp-In T-REx 293 (Thermo Fisher Scientific, Waltham, MA) are a human embryonic kidney cell line. Huh7.5.1 cells are a derivative of the Huh7 human hepatoma cella line. 293T cells are most likely female in origin (Lin et al., 2014), while Huh7 cells are male in origin (Nakabayashi et al., 1982). All three lines were maintained in DMEM containing 10% FBS and 100 U/mL penicillin-streptomycin in a37°C incubator with 5% CO2. All of these cell lines are permissive for DENV and ZIKV infection, and thus they have all of the cellular components necessary to support viral infection and replication. The initial CRISPR/Cas9 screen was performed in Huh 7.5.1 cells (Lin et al., 2017) because they exhibit marked CPE with DENV-2 infection, while 293T cells do not. Thus, the validation experiments in Figure 1 were also performed in Huh 7.5.1 cells, as were experiments with replicons. All of the transient transfection experiments were performed in 293T or HEK293 cells because they are much more efficiently transfected than Huh 7.5.1 cells.

### METHOD DETAILS

#### Plasmids, sgRNAs, and siRNAs

Individual sgRNAs were cloned into the pLENTICRISPRv2 vector for lentiviral transduction to generate EMC knockout cells (Table S1). For transient EMC knockdown, Predesigned Silencer siRNA against KIAA0090 (ID#122746, Thermo Fisher Scientific) was used as an EMC1 siRNA. AllStars negative control siRNA (QIAGEN, Hilden, Germany) was used as a scrambled siRNA.

Constructs to express epitope tagged DENV non-structural proteins were generated by PCR using DENV serotype 2 strain 16681 cDNA clone pD2/IC-30P-NBX (Huang et al., 2010), then cloned into pSMPUW (Cell Biolabs, San Diego, CA) or pCDNA4 (Thermo Fisher Scientific) expression vectors. Mutants of non-structural proteins were generated by overlap extension PCR. The TM1-lys mutant contains substitutions of L32, A35, A37, L40, A42, V43, A44, and F47 to lysine residues. The TM2-lys mutant contains substitutions of L84, I89, V91, L32, and L94 to lysine residues. The TM1-leu mutant contains substitutions of R33, P34, S36, T39, T35, T36, and T39 to leucine residues. The TM2-leu mutant contains substitutions of K80, P83, S85, K86, D88, and P92 to leucine residues. Detailed descriptions of these plasmid constructs are available upon request. A full-length infectious cDNA clone of ZIKV from the 2015 Epidemic in Brazil (ZIKV-ICD) was a generous gift from Dr. A. Pletnev (National Institutes of Health, Bethesda, MD) (Tsetsarkin et al., 2016). To construct a replication-defective cDNA clone of ZIKV, the NS5 catalytic G664-D665-D666 residues in ZIKV-ICD were mutated to G664-A665-A666 using standard cloning methods. To construct ZIKV non-structural protein expression vectors, the corresponding cDNA sequences were amplified by PCR using ZIKV-ICD as template and inserted into pcDNA3.1(−) in frame with the S tag or S-tagged GFP sequence by standard cloning methods. To construct C-terminal FLAG- or S-tagged GFP expression vectors, the GFP cDNA sequence was inserted into pcDNA3.1(−) in frame with the FLAG or Stag sequence by standard cloning methods.

Epitope tagged EMC6 and EMC4 were cloned by PCR using 293T cDNA as a template. Lentiviral expression constructs including pSMPUW and pLENTICRISPRv2 were also used to generate VSV-G pseudotyped lentiviral particles for transduction as previously described (Salloum et al., 2013).

#### Luciferase virus and replicon assays

DENV serotype 2 strain 16681 cDNA clone pD2/IC-30P-NBX was used to generate the luciferase dengue reporter virus as previously described (Lin et al., 2017). The construction of the luciferase dengue reporter replicon has also been previously reported (Wang et al., 2014). Luciferase activity was measured using the Renilla luciferase assay system (Promega, Madison, WI). The transient replicon assay was performed as previously described (Lin et al., 2017). In brief, the luciferase dengue reporter replicon was *in vitro* transcribed and 5′ capped using T7 Megascript (Thermo Fisher Scientific). RNA was then transfected into cells using TransIT mRNA reagent (Mirus Bio, Madison, WI). Luciferase activity was measured at 4 hours and 48 hours post-transfection.

#### Western blotting and band densitometry

For DENV expression constructs, cells were lysed in buffer containing 50 mM Tris pH7.5,150 mM NaCl, 1 mM EDTA, 1% SDS, and 5% glycerol, and Halt protease inhibitor (Thermo Fisher Scientific) on ice for 10 min. Lysates were clarified by centrifugation at 10k RCF for 10 min at 4°C. For NS4B, some samples were treated with PNGase F (New England Biolabs) to remove N-glycans to facilitate protein quantitation. LDS sample buffer (Thermo Fisher Scientific) was added to the lysate prior to loading the lysate on Bis-Tris NuPAGE Novex gels (Thermo Fisher Scientific).

For ZIKV expression constructs, Flp-In T-REx 293 cells (Thermo Fisher Scientific) were reverse-transfected with the indicated siRNAs at 50 μM using Lipofectamine RNAi MAX and incubated in a 12-well plate. At 48 h post siRNA transfection, cells were co-transfected with ZIKV constructs and GFP-FLAG using PEI and incubated for 24 h. Alternatively, cells were transfected with a vector containing the replication-defective ZIKV-ICD cDNA clone and incubated for 48 h. Cells were then lysed with a buffer containing 50 mM HEPES pH7.5,150 mM NaCl, 1% Triton X-100, and 1 mM PMSF, and centrifuged at 16,100 ×*g* for 10 min at 4°C. The resulting supernatants were subjected to SDS-PAGE.

After electrophoresis, proteins were transferred to a PVDF membrane, then blocked in Tris-buffered saline pH 7.5 with 0.1% Tween-20 (TBST) with 5% BSA for 30 min at room temperature. Primary antibodies were diluted in blocking buffer and incubated with the membrane at 4°C overnight. Blots were washed three times with TBST for 10 min each, incubated in HRP-conjugated secondary antibody diluted in the same blocking buffer for 1 h, and washed again three times. Bands were visualized by chemiluminescent detection with SuperSignal West Femto (Thermo Fisher Scientific) substrate in a Syngene PXi 6 (Synoptics Limited, Cambridge, UK) imager or developed using traditional X-ray film methods. Band quantification was performed using ImageJ (NIH).

#### Pulse-chase and immunoprecipitation assays

HEK293T cells (3.2 × 10^6^ cells) were reverse-transfected with the indicated siRNAs at 50 μM using Lipofectamine RNAiMAX (Thermo Fisher Scientific). At 24 h post-siRNA transfection, cells were transfected with either the NS4B-S or Derlin-1-S construct using PEI and incubated for 24 h. Cells were washed with methionine/cysteine-free cell culture medium (Thermo Fisher Scientific) and incubated at 37°C for 20 min to deplete endogenous Cys/Met. Cells were pulse-labeled with medium containing ^35^S-methionine/cysteine (^35^S-Met/Cys, 0.1 mCi/mL, Perkin Elmer, Waltham, MA), GlutaMAX (Thermo Fisher Scientific), and dialyzed FBS for 20 min at 37°C and then were collected at the indicated chase time points. The resulting whole cell lysates were incubated with S-protein agarose beads (Millipore Sigma, Darmstadt, Germany) for 2 h at 4°C to isolate S-tagged protein. The precipitated material was separated by SDS-PAGE and the radiolabeled proteins were detected by a Fujifilm phosphorimager and quantitated using ImageQuant software (GE Healthcare Life Sciences, Marlborough, MA).

For coimmunoprecipitation (co-IP) assays between the EMC and NS4B, cells were lysed in buffer containing 50 mM HEPES pH 7.5, 150 mM NaCl, 1% Deoxy Big CHAP detergent (256455, Millipore Sigma) and Halt protease inhibitor. Lysates were clarified by low speed centrifugation at 10,000 ×*g* for 10 min at 4°C. The monoclonal antibody C29F4 (Cell Signaling Technology, Danvers, MA) was added for HA immunoprecipitation. For endogenous EMC4 co-IP, an EMC4 monoclonal antibody (Abcam, Cambridge, MA) was added. After addition of antibody, samples were incubated at 4°C for 1 h. Protein G Dynabeads (Thermo Fisher Scientific) were then added, and samples were again incubated at 4°C for 1 h. Magnetically isolated beads and bound proteins were washed three times with PBS containing 0.1% Deoxy Big CHAP. Samples were eluted by addition of LDS sample buffer with 50 mM TCEP (Thermo Fisher Scientific) then heated to 95°C for 10 min.

For the sequential immunoprecipitation experiment, four 15 cm plates of Huh 7.5.1 cells stably expressing a DENV replicon encoding a puromycin resistance cassette were transfected with FLAG-Sec61β. Following incubation for 24 h, cells were lysed in 50 mM HEPES pH 7.5,150 mM NaCl, 1% Deoxy Big CHAP and 1 mM PMSF. To immunoprecipitate FLAG-Sec61β, the resulting extract was incubated with the FLAG M2 antibody-conjugated agarose beads (Sigma Aldrich) at 4°C for 2 h. After the beads were washed with the lysis buffer, bound materials were eluted with 3xFLAG peptide (Sigma Aldrich). The eluted material was then incubated with an EMC4 monoclonal antibody (Abcam) to precipitate EMC4 that had been bound to FLAG-Sec61β or with a control antibody at 4°C for 2 h. Protein G agarose beads were then added to the sample at 4°C for 1 h. After washing, the samples were subjected to SDS-PAGE and immunoblotting with the indicated antibodies.

#### Immunofluorescence microscopy

Huh 7.5.1 cells stably harboring a DENV replicon encoding puromycin resistance were stably transduced with a lentivirus encoding FLAG-EMC4 and blasticidin resistance. Cells were plated on poly-D-lysine coated coverslips then fixed in ice cold 100% methanol for 20 minutes. Coverslips were washed in PBS, then blocked in PBS with 2% BSA and 0.1% Triton X-100 for 30 minutes. The same blocking buffer was used to dilute antibodies for immunostaining, and for secondary fluorophore conjugated antibody detection.

### QUANTIFICATION AND STATISTICAL ANALYSIS

For infection and replicon experiments, each dot represents an individual biological replicate, with bars representing the mean ± SD. For band densitometry, each dot represents an individual western blot from an independent transfection. Statistical significance was determined by non-parametric Mann-Whitney U test or Dunnett’s test for multiple comparisons as indicated in the figure legends. For all figures, * p < 0.05, ** p < 0.005, and *** p < 0.0005.

## Supplementary Material

1

2

## Figures and Tables

**KEY RESOURCES TABLE:** 

REAGENT or RESOURCE	SOURCE	IDENTIFIER
Antibodies		
Anti-BAP31	Thermo Fisher Scientific	Cat# MA1–34492; RRID:AB_2537133
Anti-calnexin	Santa Cruz Biotechnology	Cat# sc-23954; RRID:AB_626783
Anti-Beta actin	Sigma Aldrich	Cat# A5316; RRID:AB_476743
Anti-DENV NS1 clone 1F11	Puttikhunt et al., 2003	
Anti-DENV NS2B	Genetex	Cat# GTX124246; RRID:AB_11170698
Anti-DENV NS4B	Genetex	Cat# GTX103349; RRID:AB_1240700
Anti-DENV NS4A	Genetex	Cat# GTX124249; RRID:AB_11177084
Anti-EMC1	Abgent	Cat# AP10226b; RRID:AB_10817224
Anti-EMC2 (TTC35)	Santa Cruz Biotechnology	Cat# Sc-166011; RRID:AB_2019521
Anti-EMC3 (TMEM111)	Santa Cruz Biotechnology	Cat# Sc-365903; RRID:AB_10842176
Anti-EMC4	Thermo Fisher Scientific	Cat# PA5–48708; RRID:AB_2634165
Anti-EMC4	Abcam	Cat# ab184162; RRID:N/A
Anti-EMC6 (TMEM93)	Aviva Systems Biology	Cat# ARP44679_P050; RRID:AB_2048477
Anti-FLAG clone M2	Sigma Aldrich	Cat# F1804; RRID:AB_262044
Anti-flavivirus E clone D1–4G2–4-15	ATCC	Cat #HB-112; RRID:CVCL_J890
Anti-GFP	Cell Signaling Technology	Cat# 2956S; RRID:AB_1196615
Anti-HA	Cell Signaling Technology	Cat# 5017S; RRID:AB_10693385
Anti-HA	Santa Cruz Biotechnology	Cat# sc-57592; RRID:AB_629568
Anti-protein disulfide isomerase	Abcam	Cat# AB2792; RRID:AB_303304
Anti-S-tag	Abcam	Cat# AB19321; RRID:AB_777789
Anti-ZIKV NS4B	Genetex	Cat# GTX133311; RRID:AB_2728825
Anti-ZIKV NS5	Genetex	Cat# GTX133312; RRID: AB_2750559
Goat anti-mouse HRP conjugated secondary	Thermo Fisher Scientific	Cat # 32430; RRID:AB_1185566
Goat anti-rabbit HRP conjugated secondary	Thermo Fisher Scientific	Cat# 32460; RRID:AB_1185567
Goat anti-mouse Alexa 488	Thermo Fisher Scientific	Cat# A-11001; RRID:AB_2534069
Goat anti-rabbit Alexa-594	Thermo Fisher Scientific	Cat# A-11037; RRID:AB_2534095
Chemicals, Peptides, and Recombinant Proteins		
4x LDS sample buffer	Thermo Fisher Scientific	NP0008
Blasticidin	Thermo Fisher Scientific	R21001
Bovine Serum Albumin	Fisher Scientific	BP1600
Deoxy Big Chap detergent	Millipore Sigma	256455
DMEM	Thermo Fisher Scientific	11995
Dynabeads Protein G	Thermo Fisher Scientific	10004D
Fetal Bovine Serum	Corning	35–010-CV
Fugene HD	Promega	E2311
G(5′)ppp(5′)A RNA Cap Structure Analog	New England Biolabs	S1406L
HALT protease inhibitor	Thermo Fisher Scientific	87786
Immobilon-P PVDF membrane	Millipore Sigma	IPVH00010
Lipofectamine RNAiMax	Thermo Fisher Scientific	13778150
MG132	Sigma Aldrich	M8699
NuPAGE 4–12% Bis-Tris Protein Gels	Thermo Fisher Scientific	NP0336BOX
Penicillin-Streptomycin	GIBCO	15140–122
PNGase F	New England Biolabs	P0704
Prolong Gold with DAPI	Thermo Fisher Scientific	P36941
Puromycin	Sigma Aldrich	P8833
Renilla Luciferase Assay System	Promega	E2820
S-protein agarose beads	Millipore Sigma	69704
SuperSignal West Femto	Thermo Fisher Scientific	34096
T7 Megascript transcription kit	Thermo Fisher Scientific	AM1334
TransIT-mRNA transfection reagent	Mirus Bio	MIR2250
Triton X-100	Sigma Aldrich	93443
Bacterial and Virus Strains		
DENV-2 strain 16681	Huang et al., 2010	N/A
ZIKV-ICD	Tsetsarkin et al., 2016	N/A
Luc-DENV	Lin et al., 2017	N/A
NEB 10-beta E. coli	New England Biolabs	C3019H
Experimental Models: Cell Lines		
Flp-In T-REx 293	Thermo Fisher Scientific	R78007
Huh 7.5.1	Zhong et al., 2005	N/A
293T	GenHunter Corp.	N/A
Recombinant DNA		
pSMPUW-IRES-Blasticidin	Cell Biolabs, Inc	VPK-219
pSPAX-2	Addgene	12260
pMD2.G	Addgene	12259
pLENTICRISPRv2	Sanjana et al., 2014	N/A
pCDNA3.1	Thermo Fisher Scientific	V79520
pCDNA4/V5-His	Thermo Fisher Scientific	V86120
Oligonucleotides		
Table S1		N/A
Software and Algorithms		
ImageJ	NIH	N/A
ImageQuant	GE Healthcare Life Sciences	N/A
Prism 7.0	GraphPad Software	N/A

## Highlights

- Dengue and Zika virus infection requires the ER membrane protein complex (EMC)
- The EMC is required for efficient expression of the viral proteins NS4A and NS4B
- The EMC dependence of NS4B is dependent on two marginally hydrophobic domains
