# Supplementary material for: The ER Membrane Protein Complex Promotes Biogenesis of Dengue and Zika Virus Non-structural Multi-pass Transmembrane Proteins to Support Infection
Source: Cell Rep. Author manuscript; Available in PMC 2019 May 16. (PMC6521869; doi:10.1016/j.celrep.2019.04.051)
Supplement: 1 [file NIHMS1528845-supplement-1.pdf]

Cell Reports, Volume *27*

Supplemental Information

The ER Membrane Protein Complex Promotes Biogenesis of Dengue and Zika Virus Non-structural Multi-pass Transmembrane Proteins to Support Infection

David L. Lin, Takamasa Inoue, Yu-Jie Chen, Aaron Chang, Billy Tsai, and Andrew W. Tai

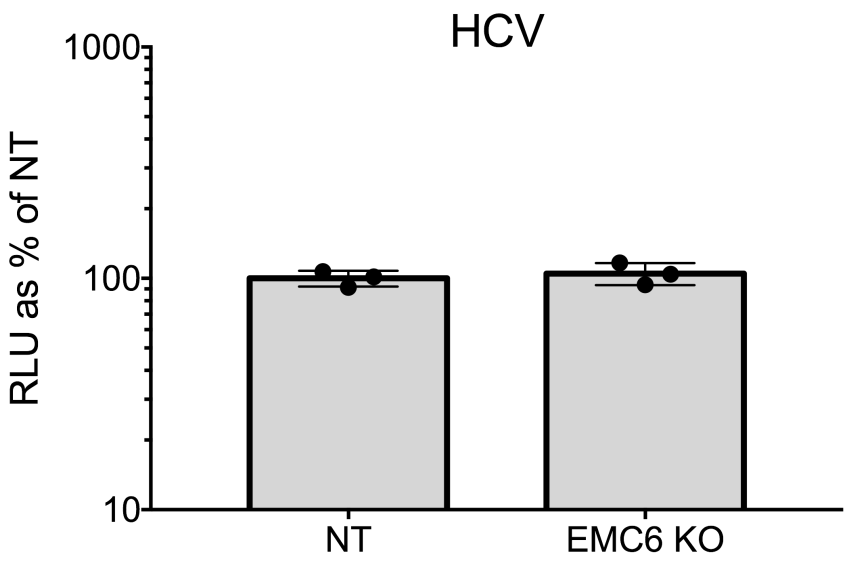

Figure S1, Related to Figure 1. Hepatitis C virus is unaffected by the loss of the EMC.

Huh 7.5.1 cells stably expressing Cas9 nuclease with either a non-targeting (NT) or EMC6 specific sgRNA were infected with HCV encoding a NanoLuc luciferase reporter followed by luciferase activity measurement 3 d later. Each dot represents a biological replicate with bars representing means±SD.

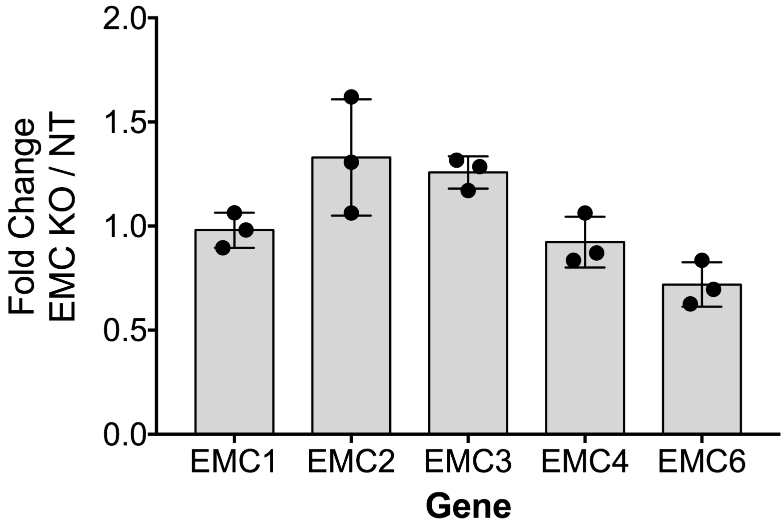

Figure S2, Related to Figure 1. Transcription of EMC subunits is unaffected by EMC6 knockout.

RNA was extracted from wild-type and EMC6 knockout Huh 7.5.1 cells, then subjected to reverse transcription and qPCR for the indicated genes. Each dot represents a biological replicate with bars showing means±SD.

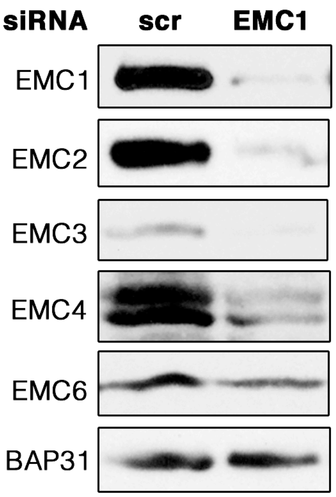

Figure S3, Related to Figure 2. EMC1 knockdown results in loss of other EMC subunits.

293 cells were transfected with a siRNA against EMC1 or scrambled control (scr) for 48 h. Cells were then lysed and subjected to SDS-PAGE and Western blotting for the indicated proteins.

Sequence	Comments
CACCG AGGCCGAATCATGCGTTCCT	F sgRNA targeting EMC1
AAAC AGGAACGCATGATTCGGCCT C	R sgRNA targeting EMC1
CACCG ACGCATGATTCGGCCTCCAT	F sgRNA targeting EMC1
AAAC ATGGAGGCCGAATCATGCGT C	R sgRNA targeting EMC1
CACCG ATACTCATTCAGCTCCCGAA	F sgRNA targeting EMC2
AAAC TTCGGGAGCTGAATGAGTAT C	R sgRNA targeting EMC2
CACCG TAATGAATATGCTTCTAAGC	F sgRNA targeting EMC2
AAAC GCTTAGAAGCATATTCATTA C	R sgRNA targeting EMC2
CACCG TGCTTGTCCAAGTAACCGAC	F sgRNA targeting EMC4
AAAC GTCGGTTACTTGGACAAGCA C	R sgRNA targeting EMC4
CACCG CAATGGCCCACTTGAAGCGC	F sgRNA targeting EMC4
AAAC GCGCTTCAAGTGGGCCATTG C	R sgRNA targeting EMC4
CACCG AATATGAACTATACCGTAAC	F sgRNA targeting EMC5
AAAC GTTACGGTATAGTTCATATT C	R sgRNA targeting EMC5
CACCG CAGGCACTTACGCTGCGCAG	F sgRNA targeting EMC5
AAAC CTGCGCAGCGTAAGTGCCTG C	R sgRNA targeting EMC5
CACCG ACGGCCGCCTCGCTGATGAA	F sgRNA targeting EMC6
AAAC TTCATCAGCGAGGCGGCCGT C	R sgRNA targeting EMC6
CACCG GACCTCGGTGTCAGCGCTGT	F sgRNA targeting EMC6
AAAC ACAGCGCTGACACCGAGGTC C	R sgRNA targeting EMC6
CACCG CCGGCAATAATCCAGGACGG	F sgRNA targeting EMC6
AAAC CCGTCCTGGATTATTGCCGG C	R sgRNA targeting EMC6

Table S1, related to Star Methods. Oligonucleotides used for sgRNA cloning into pLENTICRISPRv2 for generating EMC knockout cells. Oligos were annealed and cloned into pLENTICRISPRv2. Constructs were co-transfected into 293Ts to generate VSV-G pseudotyped lentiviruses for stable transduction of target cells.
